# Indigenous Yeasts from a Maceratino Vineyard: Sequential Fermentation as a Strategy to Enhance Wine Complexity

**DOI:** 10.3390/foods15203706

**Published:** 2026-10-18

**Authors:** Laura Moretti, Laura Canonico, Alice Agarbati, Francesca Comitini, Maurizio Ciani

**Affiliations:** Department of Life and Environmental Sciences (DiSVA), Polytechnic University of Marche, Via Brecce Bianche, 60131 Ancona, Italy; laura.moretti@pm.univpm.it (L.M.); l.canonico@univpm.it (L.C.); a.agarbati@univpm.it (A.A.); f.comitini@univpm.it (F.C.)

**Keywords:** *Metschnikowia pulcherrima*, *Lachancea thermotolerans*, mixed cultures, volatile fingerprinting, sensory profile

## Abstract

This study investigated indigenous yeasts associated with the autochthonous Maceratino grape variety (central Italy) evaluating a selection of isolates for their oenological potential and subsequent sequential fermentation trials. A total of 163 yeast isolates were collected using direct and auto-enrichment methods, of which 35 were identified with molecular methods and 16 representative strains were characterized for their oenological properties. Indigenous *Saccharomyces cerevisiae* strains showed fermentation performances comparable to commercial starters, whereas non-*Saccharomyces* species exhibited complementary technological traits despite their limited fermentative capacity. Based on the overall screening, 4 indigenous strains were selected for pure and sequential fermentation trials: *S. cerevisiae* DiSVA 898 in pure and sequential fermentation with *Metschnikowia pulcherrima* DiSVA 875, *Hanseniaspora opuntiae* DiSVA 883 and *Lachancea thermotolerans* DiSVA 890. Sequential inoculations generated wines with distinct volatile and sensory characteristics, each exhibiting a unique aromatic profile. Notably, the sequential fermentation strategy involving *L. thermotolerans* DiSVA 890 provided significant biological acidification, producing 2.16 g/L of lactic acid and an enhancement of isoamyl acetate and β-phenyl ethanol, while the sequential inoculation workflow with *M. pulcherrima* DiSVA 875 enhanced higher alcohol and ester concentrations. Overall, the results show that *S. cerevisiae* and non-*Saccharomyces* indigenous yeasts represent a valuable source for developing mixed starter cultures offering preliminary insights into the physiological potential of local isolates to modulate the analytical and sensory profile of Maceratino wine.

## 1. Introduction

Grapes and vineyard environments host complex microbial communities composed of bacteria, yeasts, and filamentous fungi. Among these microorganisms, yeasts play an important role in grape and wine production, influencing fermentation processes and the chemical and sensory characteristics of the final product [1]. The composition of grape-associated yeast communities can vary considerably depending on several factors, including geographical location, grape variety, climatic conditions, agricultural practices, and grape ripening stage [2,3]. In recent years, global warming has emerged as a major challenge in viticulture, often leading to excessive grape sugar accumulation, premature technological ripeness and a critical loss of natural acidity in musts from warm and temperate regions [4]. Although *S. cerevisiae* is the main species responsible for alcoholic fermentation, grapes and winery environments host a much wider yeast diversity [5]. Over the last decades, non-*Saccharomyces* yeasts have received increasing attention for their metabolic diversity and potential applications in winemaking and other biotechnological processes [6]. These yeasts can produce enzymes, organic acids, volatile compounds and other metabolites that may affect fermentation performance and the characteristics of the final product [7,8]. Indeed, mapping the thematic trends of non-*Saccharomyces* applications underscores their pivotal role in the deliberate synthesis of ethyl and acetate esters, which actively diversify the wine’s sensory matrix beyond standard monovarietal profiles [9].

The study of indigenous yeast populations is therefore central both to better understand the microbial ecology of vineyard and winery environments and to identify strains with potentially useful characteristics [10]. Indigenous yeasts may show considerable physiological and metabolic variability, even among strains belonging to the same species [11]. Their isolation and characterization can therefore contribute to identify strains with potential technological applications [12,13].

The selection of yeasts for oenological applications is generally based on the evaluation of several characteristics, including fermentation performance, tolerance to wine-related stress conditions, low production of undesirable metabolites and effects on the chemical and aromatic composition of wine [14,15]. However, indigenous yeasts may also possess other properties of biotechnological interest.

Vineyard and winery environments represent valuable sources of yeast biodiversity. During grape maturation and spontaneous fermentation, yeast populations undergo changes as a consequence of environmental conditions and the selective pressures associated with the fermentation process [16]. Winery surfaces and equipment may also represent additional ecological niches colonized by different yeast species [17]. The investigation of yeast populations from these environments can therefore provide information on their diversity and dynamics and allow the identification of strains with technological and functional characteristics of potential interest [18,19]. While extensive research has focused on international grape varieties, the microbial ecology and oenological potential of indigenous starter cultures associated with minor, locally adapted cultivars remain poorly explored.

The aim of the present study was to investigate the biodiversity of indigenous yeasts associated with Maceratino grapes, an autochthonous white grape variety from central Italy, and its relative winery environment. The isolated yeasts were selected and characterized for their technological and fermentative properties. The most promising strains were then employed in sequential fermentation trials to evaluate their ability to enhance acid management and aromatic complexity focusing on the impact of these selected isolates on the chemical composition and sensory characteristics of the resulting wines. Fermentation kinetics, analytical parameters, and the aromatic and sensory profiles of the resulting wines were subsequently evaluated.

## 2. Materials and Methods

### 2.1. Grape and Winery’s Environmental Sampling

The sampling campaign was carried out during the 2025 harvest season in a vineyard and winery located in Corridonia (Macerata, Italy, geographical coordinates: 43.26104624139832, 13.47429451017198). The vineyard is managed according to organic farming practices, and the winery holds an organic certification issued by Suolo e Salute (IT-BIO-004), an authorized Italian control body. The vineyard was divided into three distinct macro-areas: north, central, and south, each comprising approximately 30 rows. To ensure robust spatial independence and prevent sample cross-contamination or boundary edge effects, sampling rows were located deep within each macro-area, maintaining a minimum physical distance of eight vine rows (approximately 20 m) from one another.

A total of 10 grape samples were collected: three from the northern area, four from the central area, and three from the southern area. Within each selected row, samples were collected from distinct longitudinal positions (the beginning, middle, and end of the respective rows, spaced at least 30 m apart). Each separate collection of 3 grape bunches (approximately 1–2 kg) aseptically gathered and pooled from a unique spatial coordinate constituted a distinct biological sampling unit.

Eight of these biological sampling units belonged to the Maceratino grape variety. The remaining two biological sampling units belonged to the Montepulciano variety (a red cultivar grown within the same vineyard boundaries under identical organic management). The specific role of these Montepulciano samples was to serve as an exploratory outgroup control; they were included to evaluate whether the cultivable yeast biodiversity at harvest was primarily driven by variety-specific traits or by a shared intra-vineyard environmental baseline (the ‘vineyard effect’). To maintain a clear experimental focus on the target cultivar, these Montepulciano isolates were strictly confined to the initial cultivable taxon distribution analysis and were completely excluded from the subsequent auto-enrichment phase, as well as all down-stream micro- and sequential fermentation trials.

In addition, 5 winery environmental samples (3 samples from steel fermenters and barrels and 2 samples from floor surfaces) were collected. Samples were obtained using sterile swabs streaked over randomly selected surfaces of 10 cm^2^. The swabs were subsequently transferred into sterile tubes containing 10 mL of sterile physiological saline solution. All the samples were quickly transported to the laboratory and processed.

### 2.2. Samples Processing and Yeasts Isolation

Grape samples were separately crushed using a Stomacher homogenizer at 230 rpm for 10 min to facilitate the release of microorganisms associated with grape surfaces into the must. An aliquot of 1 mL of fresh must was decimal serially diluted and plated onto WL Nutrient Agar (Wallerstein Laboratories, Oxoid, Hampshire, UK) supplemented with 0.005% chloramphenicol to inhibit bacterial growth [20]. Plates were incubated at 25 °C for 3 days for viable yeasts enumeration and colonies isolation.

The remaining must (approximately 700 mL) and grape skins (about 150 g of wet skins) were transferred into sterile 1 L Erlenmeyer flasks locked with an airlock valve and maintained under static conditions at a controlled room temperature of 22 ± 1 °C for approximately 20 days to promote the spontaneous enrichment of the indigenous fermentative yeast population. Fermentation progress was monitored daily by measuring weight loss due to CO_2_ evolution. Samples were collected during auto-enrichment at 6 and 15 days corresponding to the middle phase (tumultuous fermentation) and the late/final phase of the process, respectively. These specific points were selected to capture the population dynamics during the active fermentation stage and the establishment of ethanol-tolerant species. The samples were subjected to viable cell counts on WL Nutrient Agar supplemented with 0.005% chloramphenicol [21].

Regarding winery environmental samples, sterile swabs were transferred into tubes containing 10 mL of sterile physiological saline solution and shaken for 1 min with vortex to promote the release of surface-associated microorganisms into the suspension. Appropriate serial decimal dilutions were carried out and plated on WL Nutrient Agar, as above described. Plates were incubated at 25 °C for 3 days [22].

After incubation, yeast colonies were selected based on differences in colony morphology. Representative colonies were isolated and purified by repeated streaking on YPD agar medium (1% yeast extract, 2% peptone, 2% glucose, 2% agar).

### 2.3. Molecular Identification of Indigenous Yeasts

A total of 163 yeast colonies were isolated and, based on colony macro- and micromorphological characteristics, 35 representative strains (at least one or two for each distinct morphotype) were selected for molecular identification. The taxonomic assignment of the remaining isolates was tentatively extended to isolates sharing the exact same macro- and micromorphological characteristics on WL Nutrient Agar. While we acknowledge that morphological grouping provides a presumptive classification and cannot completely exclude cryptic species or morphological convergence, it served as a feasible screening tool to manage the large collection. DNA was extracted according to the method described by Stringini et al. [23]. The ITS1-5.8S rRNA-ITS2 region was amplified by PCR for yeast identification following the procedure reported by Agarbati et al. [24]. Briefly, the primer set ITS1 (5′-TCCGTAGGTGAACCTCGCG-3′) and ITS4 (5′-TCCTCCGCTTTATTGATATGC-3′) was used, and the PCR products were separated by horizontal electrophoresis (Bio-Rad, Hercules, CA, USA) in a 1.5% (*w*/*v*) agarose gel using 0.5 × TBE buffer. Each yeast isolate was then identified by Sanger sequencing. All the genomic sequences obtained were compared with those already present in the data library using the BLAST program (BLAST+ v2.17.0) [25] and the GenBank database. All 35 sequenced isolates were successfully identified as yeasts, showing ≥98% query coverage and ≥99% sequence identity and they were deposited in the NCBI database under accession numbers from PZ544173 to PZ544203. Intraspecific characterization of the *S. cerevisiae* isolates was carried out using the δ12/δ21 primer pair, following the protocol described by Legras and Karst (2003) [26].

### 2.4. Preliminary Microfermentations

Sixteen strains (Table 1) were selected to carry out microfermentation trials to evaluate their fermentative aptitudes at 22 °C using fresh must composed as follows: initial sugar content 212 g/L, pH 3.47, total acidity 4.58 g/L, and yeast assimilable nitrogen (YAN) content 60 mgN/L. The sugar content was adjusted to 280 g/L adding sucrose to perform a fermentative stress test. This high sugar concentration was intentionally selected to evaluate the technical robustness and alcohol tolerance of the isolates under extreme conditions, ensuring the selection of resilient candidates. The YAN was adjusted to 250 mgN/L by the addition of diammonium phosphate [27]. Seventy mL of must were aliquoted into 100 mL flasks, pasteurized at 90–100 °C for 15 min. under controlled steam pressure to ensure microbiological sterility while minimizing thermal damage and Maillard-associated browning of the grape juice.

The strains used were precultured in YPD broth (1% *w*/*v* yeast extract, 2% *w*/*v* glucose, and 2% *w*/*v* peptone) for 48 h at 25 °C, cells were then collected, washed twice with sterile water and inoculated in 70 mL of must at an initial concentration of approximately 1 × 10^6^ cells/mL. Flasks were locked with airlock valves and incubated at 22 °C under static conditions [28].

The trials were carried out in triplicate and two commercial starter strains widely employed in the selected winery were included as controls: Alchemy II (Anchor, Oenology, Western Cape, South Africa) and Zinfandel (Enologica Vason S.p.A., Verona, Italy). An uninoculated must was included as a negative control and incubated under the same experimental conditions to account for mass loss due to evaporation.

Fermentation kinetics was monitored daily by measuring sample weight loss at 24 h intervals until a constant weight was recorded for three consecutive days. The main analytical characters, sugars content, volatile acidity and lactic acid content were determined at the end of fermentation using the standard OIV procedures [29]. Ethanol content was estimated by multiplying the total weight loss by a stoichiometric conversion factor of 1.78 [30].

### 2.5. Sequential Fermentations on Maceratino Grape Juice

Based on previous results, three strains were selected for sequential fermentation trials considering their molecular identification, fermentation performance and representativeness among the isolates tested: *M. pulcherrima* DiSVA 875, *H. opuntiae* DiSVA 883, and *L. thermotolerans* DiSVA 890. Although DiSVA 886 produced a slightly higher amount of lactic acid in the preliminary screening, DiSVA 890 was selected because it exhibited a better-balanced technological profile, combining relevant acidifying capability with a lower production of volatile acidity and a lower amount of residual sugars during microfermentations (Table 2), suggesting a more harmonious interaction in mixed cultures. Among the indigenous isolates confirmed after intraspecies characterization of isolates carried out using primer pairs δ 12/21 (Figure S1), *S. cerevisiae* strain DiSVA 898 was selected, and the commercial *S. cerevisiae* Zinfandel was used as a reference.

Pre-cultures were prepared in 10 mL of YPD broth and incubated at 25 °C for 24 h. The Pellet was then collected, washed twice with sterile water and inoculated in flasks containing 750 mL of previously pasteurized Maceratino grape must, with initial sugar content of 203.2 g/L, pH 3.61, total acidity 4.62 g/L and yeast assimilable nitrogen (YAN) content 67 mgN/L. The YAN was adjusted to 250 mgN/L by the addition of diammonium phosphate.

Five fermentation conditions were set up, two pure fermentations carried out with *S. cerevisiae* DiSVA 898 autochthonous strain and the commercial *S. cerevisiae* Zinfandel, respectively, and three sequential fermentations initially inoculated with *M. pulcherrima* DiSVA 875, *H. opuntiae* DiSVA 883 or *L. thermotolerans* DiSVA 890, followed after 72 h by the inoculation of *S. cerevisiae* DiSVA 898 autochthonous strain. All strains were inoculated at an initial concentration of 10^6^ cells/mL. Each fermentation trial was performed using two independent biological replicates, with each replicate analyzed in duplicate. Fermentation progress was monitored periodically by weight loss, until a constant weight was reached. The evolution of the yeast population was monitored by viable plate counts onto WL Nutrient Agar and Lysine agar at day of inoculation and after 3 and 7 days. Lysine Agar is a selective medium that inhibits the growth of *Saccharomyces* while allowing the growth of non-*Saccharomyces* yeasts.

#### Analytical Procedures

Ethanol content, sugar residue, volatile acidity and lactic acid were determined using the standard OIV procedures: OIV-MA-AS312-01, OIV-MA-AS311-02, OIV-MA-AS313-02, and OIV-MA-AS313-07, respectively [29]. Acetaldehyde, ethyl acetate, and higher alcohols were analyzed using a gas chromatograph system (GC-2014; Shimadzu, Kyoto, Japan) using 1 µL of sample by direct injection. Samples were injected into a 30 m × 0.32 mm column with a 0.25 μm film thickness (Zebron ZB-WAXPlus; Phenomenex, Torrance, CA, USA) using 1-pentanol (162 mg/L) as an internal standard. Helium served as carrier gas. A Shimadzu gas chromatograph (Japan) equipped with a flame ionization detector was used. The oven temperature ranged from 40 °C for 5 min, then 5 °C/min until 200 °C for 10 min, while the injector and detector temperatures were maintained at 220 °C.

The main volatile compounds were analyzed using the solid-phase microextraction (HS-SPME) method. Five mL of each sample was placed in a vial containing 1 g NaCl closed with a septum-type cap. HS-SPME was carried out under magnetic stirring for 10 min at 25 °C. After this period, an amount of 3-octanol as the internal standard (1.6 mg/L) was added, and the solution was heated to 40 °C and extracted with a Divinylbenzene/Carboxen/Polydimethylsiloxane (DVB/CAR/PDMS) fiber (Sigma-Aldrich, St. Louis, MI, USA) for 30 min by insertion into the vial headspace. The compounds were desorbed by inserting the fiber into a Shimadzu gas chromatograph GC injector for 5 min. The following glass capillary column was used: 0.25 μm Supelcowax 10 (length, 60 m; internal diameter, 0.32 mm). The fiber was inserted in split–splitless mode: 60 s splitless; the temperature of injection, 220 °C; the temperature of detector, 250 °C; carrier gas, with nitrogen; flow rate, 2.5 mL/min. The temperature program was 50 °C for 5 min, 3 °C/min to 220 °C, and then 220 °C for 20 min. The compounds were identified and quantified by comparisons with calibration curves for each compound [31].

Odor activity values (OAVs) were calculated as the ratio of each compound’s concentration to its odor threshold (OT), calculated with OTH reported by Canonico et al. and Pichler et al. [32,33], to estimate the potential contribution of individual compounds to wine aroma.

### 2.6. Sensory Analysis

At the end of fermentation and after biological stabilization, the wines were subjected to a preliminary sensory evaluation by a panel of eight expert assessors (four males and four females, aged 25–45 years), including oenologists, sommeliers and wine producers with extensive wine-tasting experience. Before the formal evaluation, the panelists participated in three calibration sessions to standardize the evaluation procedure.

Sensory evaluations were conducted under blind and randomized conditions in a standardized sensory laboratory equipped with individual booths, maintained at 22 °C, under uniform white lighting and free from interfering odors. Wines were served at 16 ± 1 °C in standard ISO tasting glasses, with 30 mL of wine per glass. Samples were presented in a randomized and counterbalanced order across assessors to minimize order and carryover effects and each wine was evaluated twice by each assessor. Mineral water and unsalted crackers were provided for palate cleansing between samples, with a 3 min break between evaluations. Each attribute was evaluated using a 10-point scale, ranging from 1 (lowest perceived intensity) to 10 (highest perceived intensity). The sensory evaluation included both olfactory attributes (ripe fruit, tropical fruit, citrus, honey, burnt toast, sweet toast, spicy, cooked vegetables, aromatic herbs, herbaceous, and phenolic notes) and gustatory attributes (acidity, alcohol perception, bitterness, softness, structure, balance, and tannicity). The individual assessor scores were analyzed using linear mixed-effects models. Informed consent was obtained from all subjects involved in the study.

### 2.7. Statistical Analysis

Statistica 7 (version 7) was used to perform analysis of variance (ANOVA) on the main analytical parameters. Analytical replicates were averaged prior to statistical analysis. For the preliminary microfermentation screening, significant differences were assessed using Duncan’s multiple range test. For the pure and sequential fermentation trials, conducted with two independent biological replicates, Tukey’s HSD test (*p* < 0.05) was used as exploratory statistical evaluation to support the descriptive comparison of analytical parameters and volatile compound concentrations [34,35].

For the sensory analysis, the two replicate evaluations performed by each assessor were averaged for each sensory descriptor. The resulting individual assessor scores were analyzed using linear mixed-effects models (LMMs), with fermentation condition as a fixed effect and assessor as a random effect to account for repeated evaluations of the different fermentation conditions by the same assessors. The effect of fermentation condition was assessed separately for each sensory descriptor, with statistical significance set at *p* < 0.05 [36].

Principal component analysis (PCA) was applied to the volatile compounds dataset to evaluate the relationships among the tested strains and to discriminate the specific chemical fingerprints generated by the different pure and sequential inoculations. PCA was performed in RStudio (v2025.05.0 + 496; RStudio Team, 2012) using R version 4.5.0.

## 3. Results

### 3.1. Isolation and Identification of Yeasts

**Figure 1:**
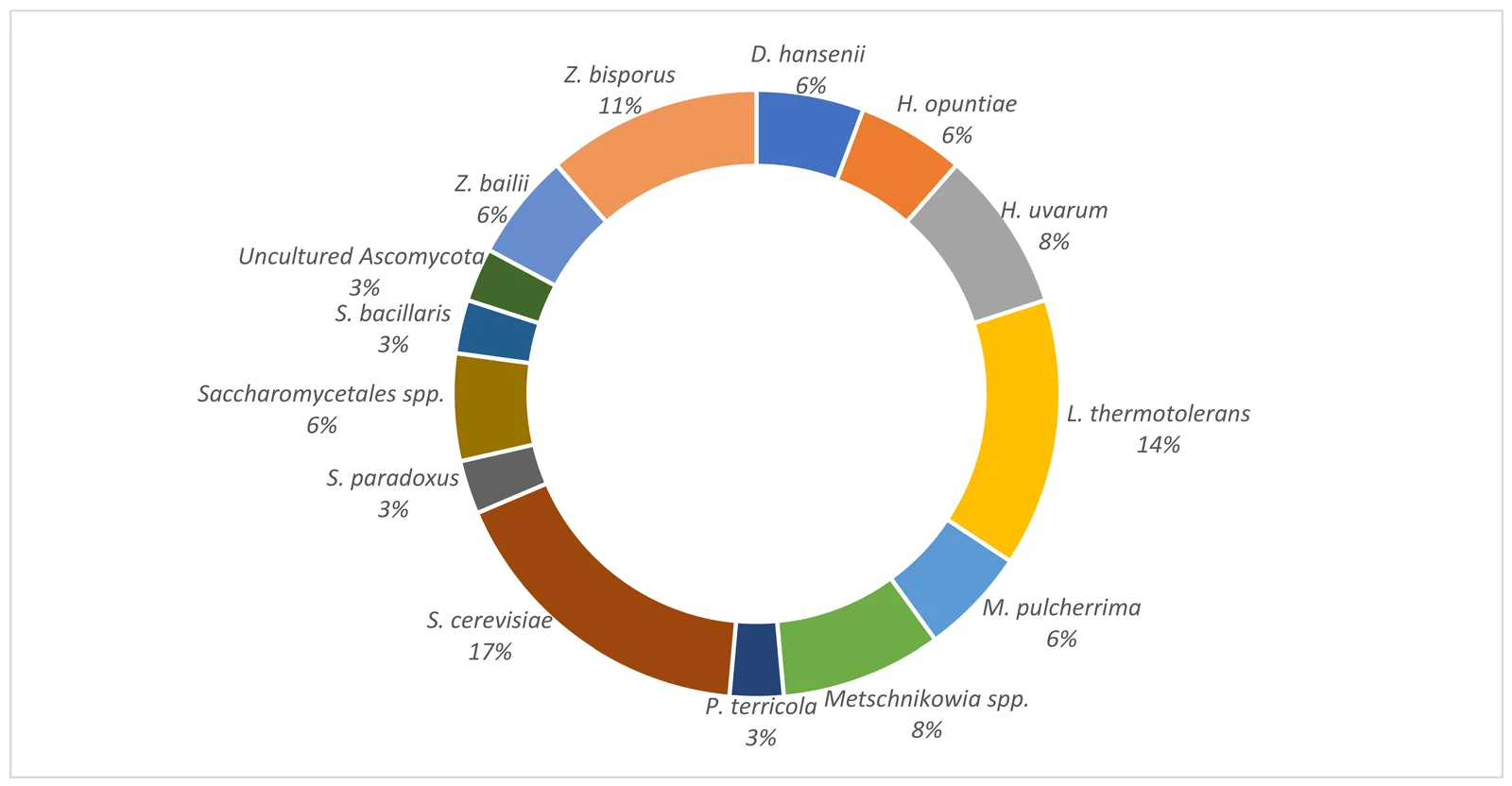
Taxonomic distribution of 35 molecolar identified yeast isolates.

A total of 163 yeast isolates were recovered from grape and winery environmental samples, 145 from grapes and 18 from environmental surfaces. Based on the similarity of their macro- and micromorphological characteristics, the isolates were grouped into homogeneous morphotypes. Representative isolates from each morphotype, 35 were subjected to ITS sequencing and molecular identification was subsequently extended to isolates sharing the same morphological characteristics. The identified collection comprised both *Saccharomyces* and non-*Saccharomyces* yeasts, including *Debaryomyces, Hanseniaspora, Lachancea, Metschnikowia, Pichia, Starmerella*, *Zygosaccharomyces* and *Saccharomyces* genera. The relative distribution of the identified taxa is reported in Figure 1, with *S. cerevisiae* (17%), *L. thermotolerans* (14%) and *Z. bisporus* (11%) representing the largest fractions of the identified collection (Figure 1).

The temporal distribution of the isolates, obtained by direct isolation and auto-enrichment, revealed a clear succession of the cultivable yeast community during spontaneous enrichment. *H. uvarum*, *H. opuntiae* and *Metschnikowia* spp. were predominantly recovered at the initial stage (direct isolation). Most of the remaining taxa were mainly isolated during the middle and final stages of fermentation following auto-enrichment. This succession was particularly evident for *Lachancea*, *Starmerella*, *Pichia*, *ZygoSaccharomyces* and *Saccharomyces* species.

### 3.2. Microfermentation Trials

Based on the molecular identification results, sixteen strains, isolated from Maceratino grapes, were selected for microfermentation trials, including all *S. cerevisiae* isolates, the unique isolate *S. paradoxus* and a representative selection of non-*Saccharomyces* species covering the taxonomic diversity recovered during the sampling campaign (Table 1). Selected strains included representatives of *M. pulcherrima*, *H. uvarum, H. opuntiae*, *L. thermotolerans*, *S. bacillaris, Z. bisporus* and *Z. bailii*. Two commercial *S. cerevisiae* starter cultures (Alchemy II and Zinfandel) were included as control.

**Figure 2:**
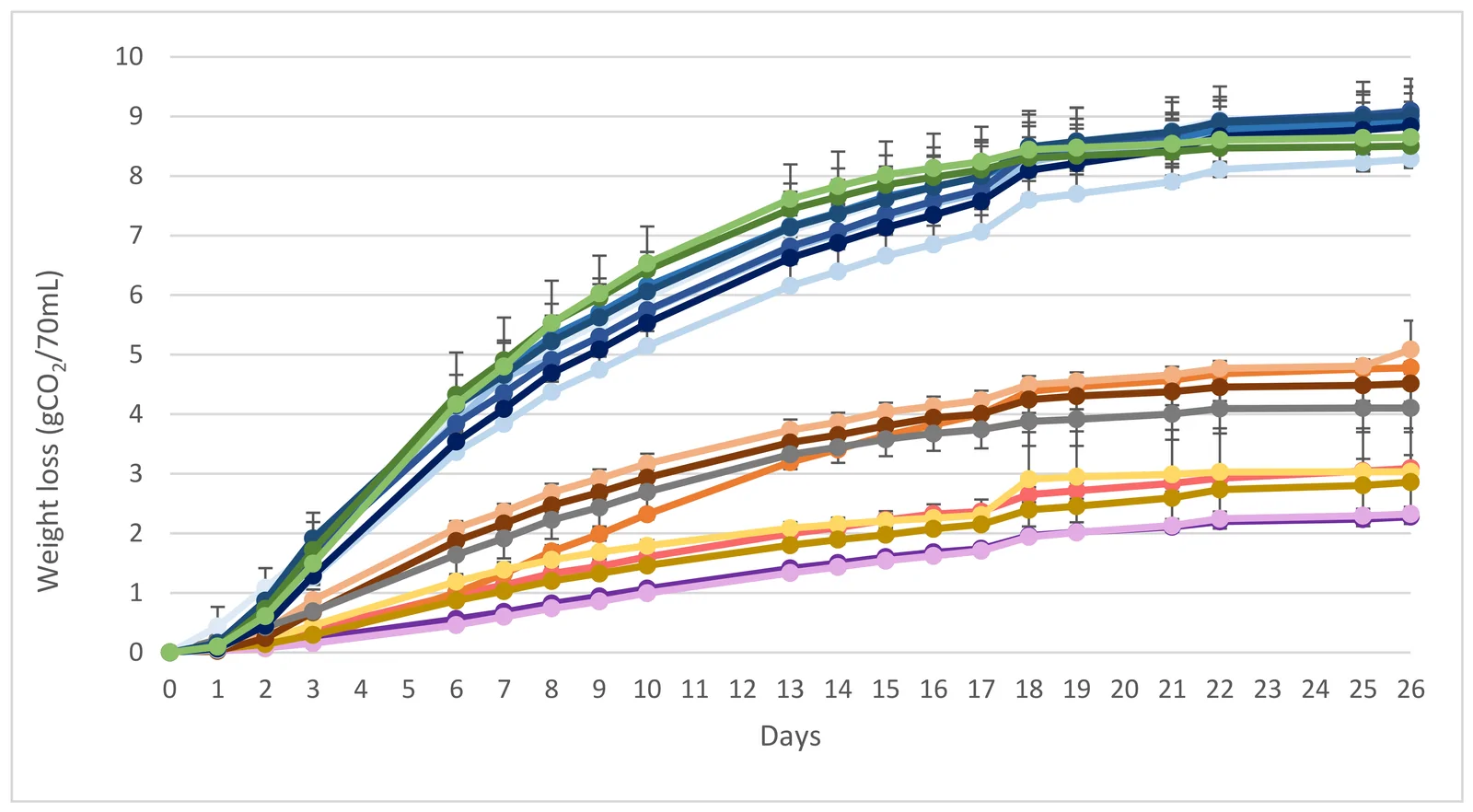
Fermentation kinetics of the yeast strains during microvinification. Yeast strains: *H. opuntiae* DiSVA 883 (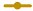), *H. uvarum* DiSVA 877 (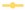), *L. thermotolerans* DiSVA 886 (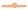), *L. thermotolerans* DiSVA 890 (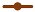), *M. pulcherrima* DiSVA 875 (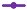), *Metschnikowia* spp. DiSVA 876 (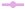), *S. bacillaris* DiSVA 870 (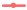), *S. cerevisiae* DiSVA 892 (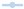), *S. cerevisiae* DiSVA 893 (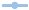), *S. cerevisiae* DiSVA 898 (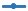), *S. cerevisiae* DiSVA 899 (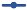), *S. cerevisiae* DiSVA 900 (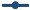), *S. cerevisiae* DiSVA 901 (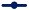), *S. paradoxus* DiSVA 891 (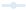), *Z. bailii* DiSVA 872 (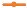), *Z. bisporus* DiSVA 897 (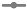), commercial *S. cerevisiae* Alchemy II (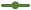), commercial *S. cerevisiae* Zinfandel (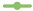).

The fermentation kinetics (Figure 2) clearly discriminated the yeast strains according to their fermentative performance (Table 2). All *S. cerevisiae* strains, including the indigenous isolates (DiSVA 892, 893, 898, 899, 900 and 901) and the commercial starters (Alchemy II and Zinfandel), exhibited the highest fermentation rates during the early stage of fermentation, resulting in a high fermentation rate reaching a plateau after approximately 18–20 days. These strains showed the highest final weight losses corresponding to ethanol concentrations between 14.75 and 16.19% (*v*/*v*) and almost complete sugar consumption, with residual sugar values below 6 g/L.

Only minor differences were observed among the *S. cerevisiae* strains. DiSVA 900 displayed the highest fermentation rate (0.64 CO_2_/day), whereas DiSVA 891 and DiSVA 898 showed fast fermentation kinetics comparable to those of commercial strains Alchemy II and Zinfandel. *S. paradoxus* (DiSVA 891) exhibited a fermentation profile almost identical to that of *S. cerevisiae*, indicating a comparable fermentative aptitude under the experimental conditions. In contrast, all non-*Saccharomyces* species displayed markedly lower fermentative performance. *L. thermotolerans* (DiSVA 886 and 890) showed the highest activity among this group, reaching ethanol concentrations of 9.05 and 8.04% (*v*/*v*), respectively, with intermediate fermentation rates (0.22–0.29 CO_2_/day). Despite their incomplete sugar consumption, these strains were characterized by relevant production of lactic acid, confirming the typical acidifying metabolism of this species. *S. bacillaris* (DiSVA 870), *Metschnikowia* spp. (DiSVA 875 and 876), and *Hanseniaspora* spp. (DiSVA 883 and 877) exhibited limited fermentation activity, with final weight losses ranging from 2.4 to 5.0 g, ethanol production below 6% (*v*/*v*), low fermentation rates (0.05–0.15 CO_2_/day), and high residual sugar concentrations (>160 g/L except for DiSVA 875). Although *Z. bailii* (DiSVA 872) and *Z. bisporus* (DiSVA 897) produced relatively higher ethanol concentrations (8.51 and 7.31% *v*/*v*, respectively), their fermentation rates remained substantially lower than those of *Saccharomyces*, and fermentations stopped with more than 110 g/L residual sugars.

Overall, the fermentation kinetics were consistent with the analytical data, confirming the clear superiority of *Saccharomyces* strains in terms of fermentation rate, ethanol production and sugar utilization. Conversely, the non-*Saccharomyces* species were unable to complete alcoholic fermentation under the tested conditions, although *L. thermotolerans* distinguished itself by its remarkable lactic acid production.

### 3.3. Sequential Fermentation Trials

**Figure 3:**
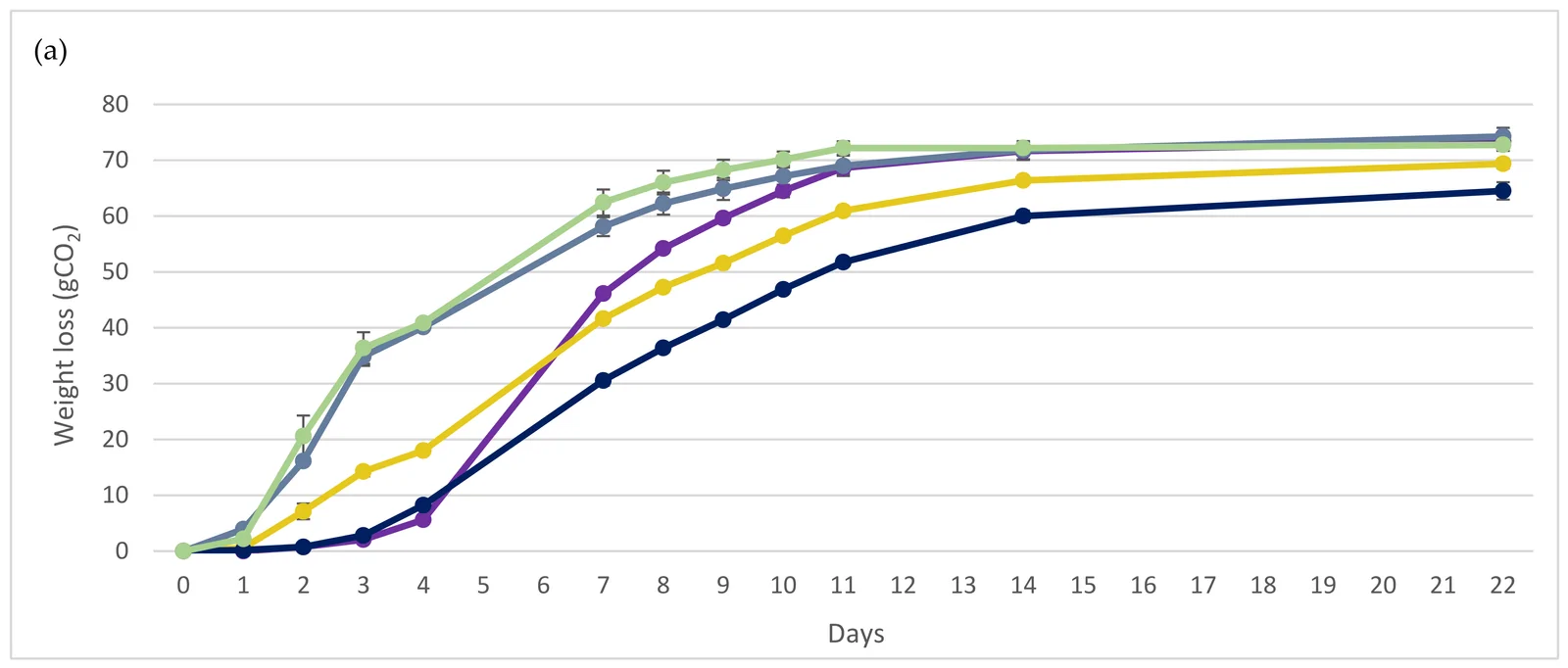

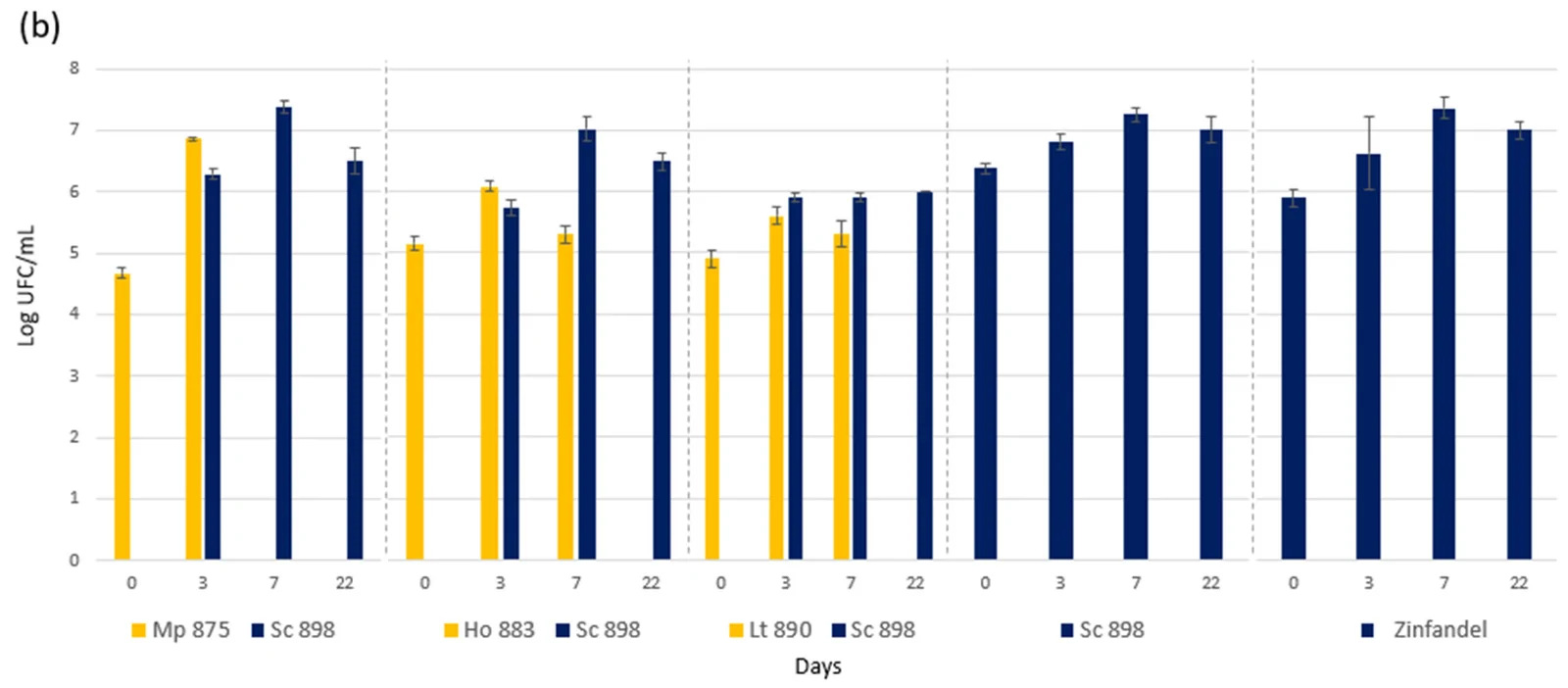
Fermentation kinetics (**a**) and growth kinetics (**b**) of wines produced by sequential fermentation: *M. pulcherrima* DiSVA 875/*S. cerevisiae* DiSVA 898 (Mp875/Sc898 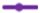); *H. opuntiae* DiSVA 883/*S. cerevisiae* DiSVA 898 (Ho883/Sc898 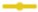) and *L. thermotolerans* DiSVA 890/*S. cerevisiae* DiSVA 898 (Lt890/Sc898 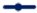) and pure fermentation: *S. cerevisiae* DiSVA 898 (Sc898 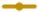), and the commercial starter *S. cerevisiae* Zinfandel (Zinfandel 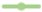).

The fermentation kinetics and population dynamics of *Saccharomyces* and non-*Saccharomyces* yeasts are shown in Figure 3. The two pure *S. cerevisiae* fermentations exhibited comparable fermentation profiles, reaching approximately 70 g CO_2_ lost after 14 days. During the first three days of the sequential fermentations, the non-*Saccharomyces* strains reached high population densities, while *S. cerevisiae* DiSVA 898 was not detected, in accordance with the inoculation strategy. Following inoculation of the indigenous *S. cerevisiae* strain at day 3, fermentation rates increased in all sequential trials, although with different kinetics depending on the non-*Saccharomyces* species. The fermentation started with *M. pulcherrima* DiSVA 875 showed the most rapid increase in CO_2_ production after the addition of *S. cerevisiae* DiSVA 898, whereas fermentations started with *H. opuntiae* DiSVA 883 and *L. thermotolerans* DiSVA 890 progressed more slowly and required a longer time to reach the final CO_2_ production. This behaviour was reflected in the yeast population dynamics. By day 7, *M. pulcherrima* DiSVA 875 was no longer detectable, while *S. cerevisiae* DiSVA 898 had become the only detectable yeast population. In contrast, both *H. opuntiae* DiSVA 883 and *L. thermotolerans* DiSVA 890 remained viable at day 7, coexisting with *S. cerevisiae* DiSVA 898 throughout the monitored period. This population shift, accurately quantified by the viable plate counts shown in Figure 3b, underscores the different competitive fitness and tolerance levels of non-*Saccharomyces* species under increasing ethanol concentrations. Despite these differences in fermentation kinetics and yeast persistence, all fermentation conditions reached comparable cumulative CO_2_ production at the end of the experiment. In the control fermentations, *S. cerevisiae* maintained consistently high cell densities throughout the entire fermentation process.

### 3.4. Main Analytical Parameters

In all wines, despite the different inoculated yeasts among treatments, comparable ethanol concentrations and low residual sugar levels (below 0.04 g/L) were observed (Table 3). Regarding the other analytical parameters, only the sequential fermentation *L. thermotolerans* DiSVA 890/*S. cerevisiae* DiSVA 898 showed consistent and significant lactic acid production and a moderate increase in acetic acid.

### 3.5. Main Volatile Compounds

Results of main volatile compounds of sequential fermentation trials are shown in Table 4. The presence of non-*Saccharomyces* strains in sequential fermentation was associated with several differences in the volatile composition of the wines, particularly the ester and higher alcohol fractions. Among them, the sequential fermentation strategy of *L. thermotolerans* DiSVA 890/*S. cerevisiae* DiSVA 898 was characterized by the highest concentrations of isoamyl acetate and ethyl acetate, together with high acetaldehyde levels, increased levels of β-phenyl ethanol, *1*-propanol and isobutanol. In contrast, sequential fermentation with *H. opuntiae* DiSVA 883 resulted in the highest ethyl butyrate and isoamyl alcohol concentrations, whereas *M. pulcherrima* DiSVA 875/*S. cerevisiae* DiSVA 898 was associated with the highest linalool and comparatively high β-phenyl ethanol levels. Wines produced with the commercial Zinfandel strain generally showed intermediate volatile profiles, while the pure fermentation with *S. cerevisiae* DiSVA 898 was characterized by the highest ethyl hexanoate and acetaldehyde concentration and among the lowest levels of ethyl acetate.

**Figure 4:**
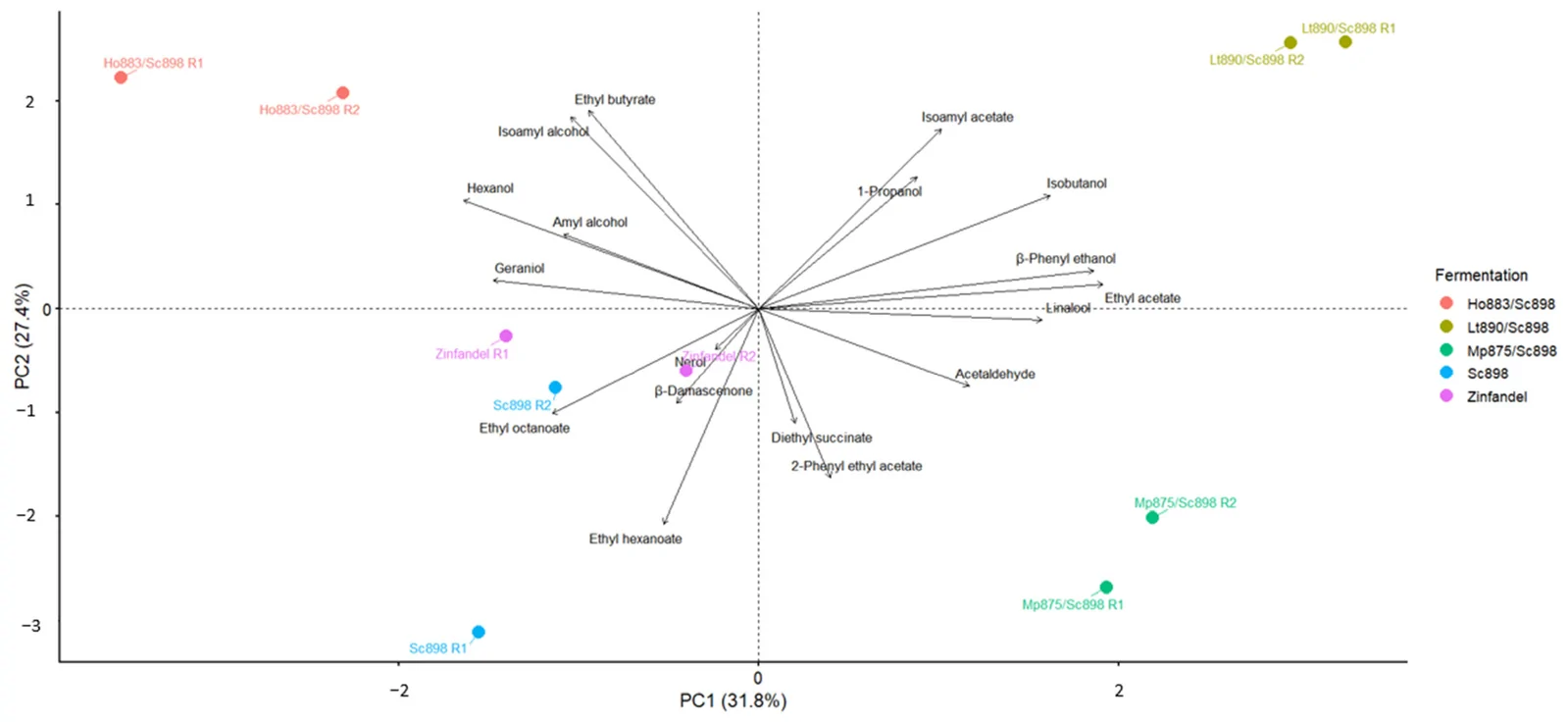
Principal component analysis (PCA) biplot of volatile compounds and higher alcohols of pure fermentation of *S. cerevisiae* DiSVA 898 (Sc898), commercial strain Zinfandel and sequential fermentations of *M. pulcherrima* DiSVA 875 (Mp875_Sc898), *H. opuntiae* DiSVA 883 (Ho883_Sc898) and *L. thermotolerans* DiSVA 890 (Lt890_Sc898). R1 and R2 indicate the two independent biological replicates.

The main volatile compounds were analyzed by principal component analysis (PCA) to explore differences among fermentation strategies, with each biological replicate represented as a separate observation using the mean of two analytical replicates (Figure 4).

The first two principal components explained 59.2% of the total variance, with PC1 and PC2 accounting for 31.8% and 27.4%, respectively. The biplot showed a clear separation of the wines according to the fermentation strategy, indicating distinct volatile profiles among pure and sequential fermentations. The two pure *S. cerevisiae* fermentations were positioned on the negative side of PC1, without a clear separation between the two conditions. The Zinfandel replicates showed limited dispersion along PC2, whereas those of *S. cerevisiae* DiSVA 898 were more widely separated along this axis. In particular, *S. cerevisiae* DiSVA 898 Replicate 1 (R1) extended further towards the ethyl hexanoate vector, while Replicate 2 (R2) was positioned closer to the Zinfandel samples, in the direction of ethyl octanoate and β-damascenone. This distribution highlights variability between the *S. cerevisiae* DiSVA 898 replicates and suggests caution in assigning a distinct volatile profile to either pure fermentation based on the first two principal components alone. The sequential fermentations occupied different regions of the biplot, with both replicates of each condition positioned in the same quadrant. *M. pulcherrima* DiSVA 875/*S. cerevisiae* DiSVA 898 was located on the positive side of PC1 and negative side of PC2, mainly in the direction of linalool, 2-phenyl ethyl acetate, diethyl succinate and acetaldehyde. *H. opuntiae* DiSVA 883/*S. cerevisiae* DiSVA 898 was associated with ethyl butyrate, isoamyl alcohol, hexanol, geraniol and amyl alcohol. In contrast, *L. thermotolerans* DiSVA 890/*S. cerevisiae* DiSVA 898 was clearly separated on the positive side of both PC1 and PC2 and was mainly associated with isoamyl acetate, 1-propanol, isobutanol and ethyl acetate. Overall, the PCA showed that each non-*Saccharomyces* species in sequential fermentation generated a distinct volatile fingerprint, consistent with the differences observed in the quantitative volatile analysis.

### 3.6. Sensory Evaluation

**Figure 5:**
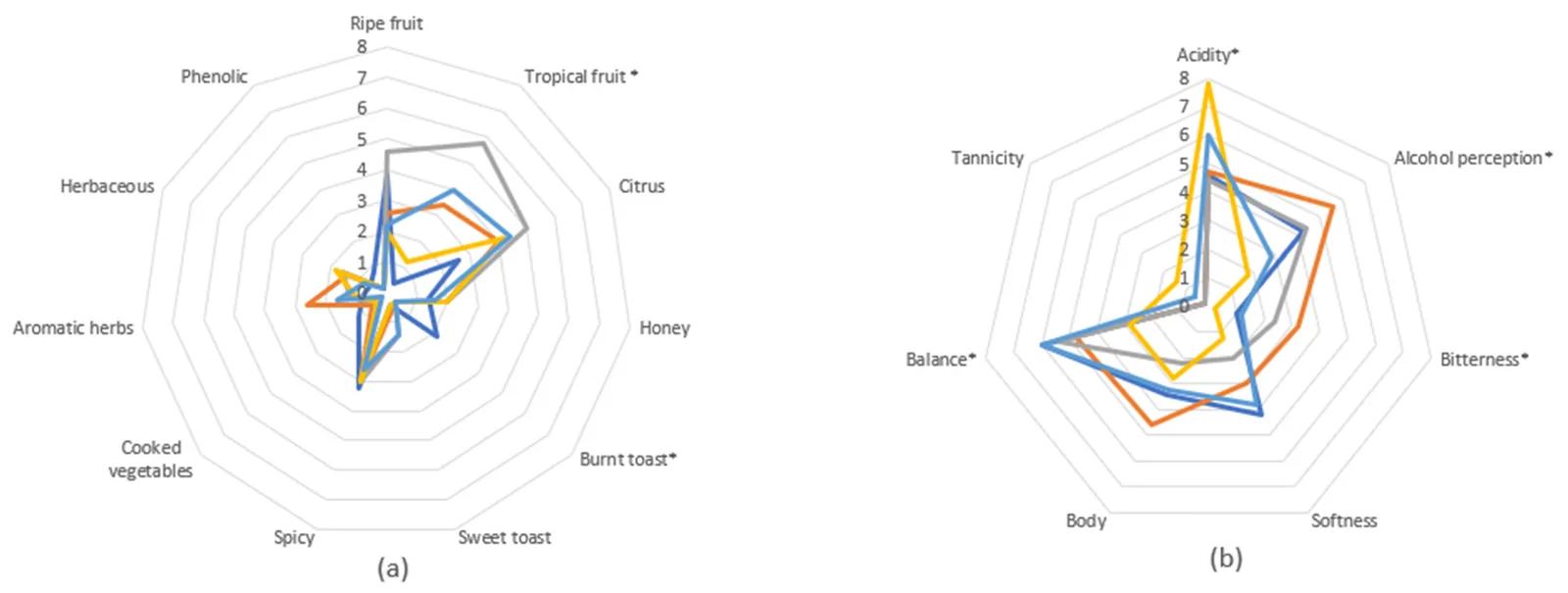
Sensory analysis of wines produced in pure and sequential fermentations: (**a**) olfactory analysis and (**b**) taste analysis. *S. cerevisiae* DiSVA 898 (Sc 898 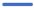), *S. cerevisiae* Zinfandel (Zinfandel 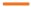), *M. pulcherrima* DiSVA 875/*S. cerevisiae* DiSVA 898 (Mp 875/Sc 898 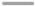), *L. thermotolerans* DiSVA 890/*S. cerevisiae* DiSVA 898 (Lt 890/Sc 898 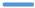), *H. opuntiae* DiSVA 883/*S. cerevisiae* DiSVA 898 (Ho 883/Sc 898 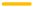). The results were analyzed using linear mixed-effects models (LMMs) (* *p* < 0.05).

Sensory evaluation highlighted distinct sensory profiles among the wines obtained under the different fermentation strategies (Figure 5a,b). The wine produced by sequential fermentation with *L. thermotolerans* DiSVA 890 was characterized by pronounced tropical fruit and citrus aromas, enhanced perceived acidity and softness, and an overall balanced sensory profile with reduced alcohol perception. The *H. opuntiae* DiSVA 883/*S. cerevisiae* DiSVA 898 fermentation was mainly associated with citrus and spicy notes, while exhibiting the highest acidity and lower body and alcohol perception than pure *S. cerevisiae* fermentations. The wine obtained with *M. pulcherrima* DiSVA 875/*S. cerevisiae* DiSVA 898 showed the most pronounced ripe fruit and tropical fruit characters, accompanied by intermediate scores for most gustatory descriptors, including acidity, softness and body.

The two wines produced by pure *S. cerevisiae* fermentations exhibited overall similar sensory profiles. However, the indigenous *S. cerevisiae* DiSVA 898 strain received slightly higher scores for ripe fruit, burnt toast, spicy notes, softness and balance perception than the commercial Zinfandel strain, whereas both wines showed comparable perceptions of acidity. Overall, sequential inoculation with different non-*Saccharomyces* species generated wines with clearly distinguishable sensory characteristics, mainly affecting fruity aroma descriptors and taste perception.

## 4. Discussion

This study integrates the ecological characterization of indigenous yeasts associated with Maceratino grapes with a technological evaluation of selected isolates to assess their potential in winemaking. Starting from a collection of 163 cultivable yeasts recovered from grape berries and winery surfaces, 35 representative isolates were identified using molecular methods and subsequently screened for their fermentative and biotechnological properties, allowing the selection of the most promising strains for sequential fermentation trials. This approach reflects the current trend in wine microbiology, where indigenous yeast biodiversity is increasingly regarded not only as an ecological feature of vineyards but also as a valuable reservoir of functional traits with potential applications in winemaking and wine quality improvement [10,11,37,38,39].

The identified yeast pool, using direct and auto-enrichment approaches, confirmed the biodiversity typically associated with grape berries, including both *Saccharomyces* and non-*Saccharomyces* genera [40]. Although the composition of grape-associated yeast communities is known to vary according to vine variety, geographical origin, climatic conditions and vineyard management, *Hanseniaspora* and *Metschnikowia* are consistently reported among the dominant genera on freshly harvested grapes [41,42,43]. The wide presence of *M. pulcherrima*, negatively affected by the organic treatments, indicated the low level of treatments (close to absence) in this organic winery [24]. This specific distribution highlights how conservative agricultural practices can safeguard the natural yeast community.

In agreement with these observations, most isolates recovered with direct isolation (T0) belonged to these two genera, whereas species belonging to *Lachancea*, *Starmerella*, *Pichia*, *Zygosaccharomyces* and *Saccharomyces* were mainly isolated after 6 and 15 days of spontaneous auto-enrichment [44,45,46]. The results of this work confirm that *S. cerevisiae* is isolated with extreme difficulty from natural substrates such as the surface of mature grapes [47]. Under auto-enrichment conditions, with the progressive establishment of fermentation (increasing ethanol concentration, nutrient depletion and oxygen availability) *S. cerevisiae* and other fermenting species take over and may be easily isolated [48].

Based on taxonomic diversity and winemaking aptitude, sixteen isolates were selected for technological characterization. All indigenous *S. cerevisiae* strains showed fermentation kinetics and fermentation power comparable with those of the two commercial starter cultures, completing alcoholic fermentation (must with high sugar content) with almost complete sugar consumption. As expected, the non-*Saccharomyces* isolates displayed slower fermentation rates and higher residual sugar contents, confirming their limited fermentative capacity when used individually. Among these, the two *L. thermotolerans* isolates were distinguished by their higher lactic acid production, consistently with the well-established acidifying ability of this species and its increasing interest for improving freshness and acidity in wines produced from warm-climate grapes [49,50].

Non-*Saccharomyces* wine species have attracted growing interest as complementary starters in mixed fermentations that contribute with specific metabolic activities [51,52,53]. Their technological value therefore resides in their capacity to diversify wine composition through the production of enzymes, organic acids and aroma-active metabolites that are often absent or less pronounced in pure *Saccharomyces* fermentations [54,55]. Accordingly, sequential inoculation has become one of the most effective strategies for combining the fermentative robustness of indigenous *S. cerevisiae* with the metabolic potential of non-*Saccharomyces* yeasts [56,57]. In line with this approach, the three indigenous non-*Saccharomyces* strains were assayed for sequential fermentation with the indigenous *S. cerevisiae* DiSVA 898 that exhibited fermentation performance comparable to those of the commercial starter. While pure fermentations (commercial and indigenous *S. cerevisiae* strains) showed comparable results, sequential fermentations exhibited improved complexity and well-characterized wines [58]. In this regard, non-*Saccharomyces* indigenous yeasts represented a valuable source for developing mixed starter cultures capable of enhancing wine complexity preserving the microbial population of the winemaking area. *M. pulcherrima*/*S. cerevisiae* sequential fermentation showed a limited fermentative activity during the first three days, followed by a rapid increase in fermentation rate after *S. cerevisiae* inoculation. Nevertheless, its early metabolic activity was associated with a distinct volatile composition of the resulting wine. As showed by the multivariate PCA, the sequential fermentation with *M. pulcherrima* was effectively separated from the other trials primarily due to the higher concentrations of 2-phenyl ethyl acetate, acetaldehyde and linalool (OAVs > 1) associated with increased sensory perception of tropical and ripe fruit aromas and with good overall balance [59,60]. This phenomenon may be related to the presence of *M. pulcherrima* during the first 72 h of fermentation, potentially contributing to aroma release through extracellular enzymes such as β-glucosidases before its population declined [33]. The *H. opuntiae/S. cerevisiae* sequential fermentation was characterized by a longer persistence of *H. opuntiae*-The interactions of this sequential fermentation determining a volatile profile characterized by higher concentrations of ethyl butyrate and isoamyl alcohol with OAVs > 1. Despite these compositional differences, the sensory profile was mainly distinguished by a greater perception of acidity rather than enhanced fruity attributes, suggesting that changes in volatile composition were only partially reflected in aroma perception [61]. Similarly, *L. thermotolerans* remained detectable after *S. cerevisiae* inoculation. This sequential fermentation showed the most differentiated metabolic profile. In addition to lactic acid production, this wine exhibited the highest concentrations of isoamyl acetate, ethyl acetate and β-phenyl ethanol, all showing OAVs > 1, as well as increased levels of 1-propanol, acetaldehyde and isobutanol, contributing to aclear separation in the PCA. Sensory evaluation also highlighted greater softness together with good overall balance [62]. These results are of particular oenological relevance for the technological characterization of local white varieties. The organic acid production, specifically the biological synthesis of lactic acid demonstrated by the indigenous *L. thermotolerans* strains under sequential fermentation conditions, represents a potential natural tool to modulate wine acidity, while simultaneously enhancing the ester-driven aromatic complexity of the final product. These metabolic outcomes align with previous investigations on *L. thermotolerans* strains from the DiSVA collection, which demonstrated a capacity to upregulate acetate esters and higher alcohols, thereby potentially improving the structural softness and olfactory balance of the matrix [63]. In this context, the literature suggests that such lactic acid production could be highly beneficial under warm-climate conditions where organic acids are naturally low [57]. The volatile and acid modulations must be interpreted as the combined metabolic outcome of the sequential fermentation process. This includes early interactions during the first 72 h, such as nutrient depletion or enzyme release by the non-*Saccharomyces* isolate, which sequentially redirect the metabolic behavior of the subsequently inoculated *S. cerevisiae* DiSVA 898.

Overall, the present study indicated that the technological differences observed among the sequential fermentations were associated more on their species-specific metabolic activity during the initial phases than on their long-term persistence throughout the entire fermentation process [64]. Interestingly, the persistence of the inoculated non-*Saccharomyces* species was not directly related to their overall technological impact. *M. pulcherrima* rapidly disappeared after *S. cerevisiae* inoculation but still generated substantial modifications in wine aroma composition. This observation suggests that transient metabolic activity during the early stages of fermentation may be sufficient to redirect yeast metabolism and influence the final volatile profile [65,66]. This could be associated with species-dependent amino acid depletion kinetics during the first 72 h; by altering the available nitrogen pool, non-*Saccharomyces* starters may induce a metabolic shift in the subsequently inoculated *S. cerevisiae*, stimulating the synthesis of volatile higher alcohols [67]. These outcomes provide a promising proof-of-concept regarding the functional potential of local isolates. While these findings demonstrate distinct volatile fingerprints among sequential fermentations, any link to ‘microbial terroir’ or definitive ‘territorial expression’ must be treated strictly as a working hypothesis. True validation of a regional microbial signature demands multi-vintage monitoring across diverse sub-zones to separate genetic strain stability from seasonal climatic fluctuations. Nevertheless, our data provide a valuable baseline showing that local *non-Saccharomyces* isolates possess specific technological assets that merit further investigation as potential tools for preserving regional oenological diversity [68]. Overall, this study highlights the potential of integrating ecological characterization with technological evaluation to identify indigenous non-*Saccharomyces* yeasts suitable for sequential fermentations [69,70,71].

## 5. Conclusions

Overall, the yeast diversity recovered from Maceratino grapes and the winery environment provides a valuable basis for evaluating mixed culture formulations under laboratory conditions. In these preliminary trials, sequential fermentations involving specific local isolates generated distinctive volatile and sensory profiles. Particularly, the sequential inoculation with *Lachancea thermotolerans* DiSVA 890 showed promising technological traits due to its specific capability to produce lactic acid, which represents a potential biological tool for acid management in musts. However, further studies spanning multiple vintages and pilot-scale trials are strictly required to validate the stability, reproducibility, and industrial viability of these local isolates before any definitive conclusion regarding their contribution to regional wine profiles can be established.

## Figures and Tables

**Table 1:** Yeast strains selected for microfermentation trials.

DiSVA Code	Species	Isolation Source	DiSVA Code	Species	Isolation Source
DiSVA 883	*H. opuntiae*	Maceratino grape	DiSVA 898	*S. cerevisiae*	Maceratino grape
DiSVA 877	*H. uvarum*	Maceratino grape	DiSVA 899	*S. cerevisiae*	Maceratino grape
DiSVA 886	*L. thermotolerans*	Maceratino grape	DiSVA 900	*S. cerevisiae*	Maceratino grape
DiSVA 890	*L. thermotolerans*	Maceratino grape	DiSVA 901	*S. cerevisiae*	Maceratino grape
DiSVA 875	*M. pulcherrima*	Maceratino grape	DiSVA 891	*Saccharomyces paradoxus*	Maceratino grape
DiSVA 876	*Metschnikowia* spp.	Fermentation tank	DiSVA 872	*ZygoSaccharomyces bailii*	Maceratino grape
DiSVA 870	*Starmerella bacillaris*	Maceratino grape	DiSVA 897	*ZygoSaccharomyces bisporus*	Maceratino grape
DiSVA 892	*S. cerevisiae*	Maceratino grape	Alchemy II	*S. cerevisiae*	Commercial starter culture
DiSVA 893	*S. cerevisiae*	Maceratino grape	Zinfandel	*S. cerevisiae*	Commercial starter culture

**Table 2:** Fermentation analytical parameters. Data are means ± standard deviations. Values displaying different superscript letters (^a,b,c,d,e,f,g,h,i^) within each column are significantly different according to Duncan’s tests (*p* < 0.05). * In the first three days.

DiSVA Code	Weight Loss (g/70 mL)	Fermentation Rate (CO_2_/Day *)	Ethanol %/vol.	Residual Sugar (g/L)	Volatile Acidity (g/L)	Lactic Acid (g/L)
DiSVA 883	2.86 ± 0.01 ^abcd^	0.10 ± 0.01 ^efg^	5.09 ± 0.03 ^bc^	165.44 ± 1.25 ^a^	0.16 ± 0.05 ^abcd^	0.04 ± 0.01 ^ghi^
DiSVA 877	3.04 ± 0.73 ^bc^	0.15 ± 0.03 ^defg^	5.40 ± 1.30 ^bc^	170.11 ± 8.22 ^a^	0.20 ± 0.04 ^ab^	0.04 ± 0.01 ^gh^
DiSVA 886	5.08 ± 0.49 ^b^	0.29 ± 0.02 ^cd^	9.05 ± 0.87 ^b^	121.23 ± 2.68 ^bc^	0.07 ± 0.00 ^fg^	2.92 ± 0.02 ^a^
DiSVA 890	4.52 ± 0.30 ^bc^	0.22 ± 0.01 ^def^	8.04 ± 0.54 ^bc^	138.03 ± 7.15 ^b^	0.07 ± 0.00 ^fg^	2.24 ± 0.01 ^b^
DiSVA 875	2.28 ± 0.00 ^b^	0.08 ± 0.00 ^fg^	4.06 ± 0.00 ^b^	128.69 ± 0.71 ^c^	0.05 ± 0.00 ^efg^	0.03 ± 0.01 ^ghi^
DiSVA 876	2.33 ± 0.12 ^c^	0.05 ± 0.01 ^g^	4.14 ± 0.21 ^c^	173.02 ± 5.89 ^a^	0.06 ± 0.01 ^g^	0.02 ± 0.00 ^i^
DiSVA 870	3.09 ± 0.23 ^bc^	0.13 ± 0.01 ^efg^	5.50 ± 0.40 ^bc^	170.11 ± 13.93 ^a^	0.17 ± 0.04 ^abcd^	0.05 ± 0.01 ^fg^
DiSVA 892	8.29 ± 0.15 ^a^	0.42 ± 0.02 ^bc^	14.75 ± 0.26 ^a^	5.58 ± 0.16 ^d^	0.17 ± 0.00 ^abc^	0.17 ± 0.01 ^d^
DiSVA 893	8.85 ± 0.26 ^a^	0.55 ± 0.04 ^ab^	15.76 ± 0.47 ^a^	4.30 ± 0.33 ^d^	0.14 ± 0.00 ^bcde^	0.06 ± 0.02 ^f^
DiSVA 898	8.92 ± 0.47 ^a^	0.58 ± 0.20 ^ab^	15.87 ± 0.84 ^a^	4.56 ± 0.04 ^d^	0.14 ± 0.00 ^bcde^	0.18 ± 0.00 ^d^
DiSVA 899	9.09 ± 0.40 ^a^	0.56 ± 0.09 ^ab^	16.19 ± 0.72 ^a^	5.38 ± 1.33 ^d^	0.16 ± 0.02 ^abcd^	0.18 ± 0.01 ^d^
DiSVA 900	9.02 ± 0.23 ^a^	0.64 ± 0.09 ^a^	16.06 ± 0.40 ^a^	4.76 ± 0.63 ^d^	0.13 ± 0.01 ^cde^	0.21 ± 0.00 ^c^
DiSVA 901	8.83 ± 0.67 ^a^	0.43 ± 0.05 ^bc^	15.73 ± 1.20 ^a^	5.07 ± 0.44 ^d^	0.21 ± 0.02 ^a^	0.14 ± 0.01 ^e^
DiSVA 891	9.08 ± 0.55 ^a^	0.58 ± 0.08 ^ab^	16.16 ± 0.98 ^a^	4.07 ± 0.01 ^d^	0.17 ± 0.00 ^abc^	0.12 ± 0.02 ^e^
DiSVA 872	4.78 ± 0.10 ^b^	0.07 ± 0.03 ^efg^	8.51 ± 0.18 ^b^	123.50 ±3.0 ^bc^	0.07 ± 0.00 ^fg^	0.03 ± 0.00 ^hi^
DiSVA 897	4.11 ± 0.40 ^bc^	0.23 ± 0.12 ^de^	7.31 ± 0.72 ^bc^	114.03 ±8.2 ^bc^	0.08 ± 0.04 ^fg^	0.16 ± 0.03 ^d^
Alchemy II	8.50 ± 0.23 ^a^	0.57 ± 0.01 ^ab^	15.14 ± 0.42 ^a^	3.24 ± 0.01 ^d^	0.11 ± 0.01 ^defg^	0.19 ± 0.01 ^cd^
Zinfandel	8.65 ± 0.40 ^a^	0.50 ± 0.00 ^ab^	15.40 ± 0.70 ^a^	3.47 ± 0.44 ^d^	0.12 ± 0.00 ^cdef^	0.15 ± 0.01 ^e^

**Table 3:** Analytical parameters of sequential fermentation: *M. pulcherrima* DiSVA 875/*S. cerevisiae* DiSVA 898 (Mp875/Sc898); *H. opuntiae* DiSVA 883/*S. cerevisiae* DiSVA 898 (Ho883/Sc898) and *L. thermotolerans* DiSVA 890/*S. cerevisiae* DiSVA 898 (Lt890/Sc898) and pure fermentation: *S. cerevisiae* DiSVA 898 (Sc898) and the commercial starter *S. cerevisiae* Zinfandel. Data are means ± standard deviations. Values displaying different superscript letters (^a,b,c^ within each column are significantly different according to Tukey’s HSD test (*p* < 0.05).

Parameter	DiSVA Code	Ethanol (% *v*/*v*)	Sugars (g/L)	Lactic Acid (g/L)	Acetic_Acid (g/L)	pH
***H. opuntiae*/*S. cerevisiae***	883/898	12.52 ± 0.25 ^a^	0.02 ± 0.00 ^b^	0.02 ± 0.00 ^b^	0.12 ± 0.00 ^b^	3.42 ± 0.00 ^a^
***L. thermotolerans*/*S. cerevisiae***	890/898	12.89 ± 0.10 ^a^	0.00 ± 0.00 ^c^	2.16 ± 0.09 ^a^	0.23 ± 0.01 ^a^	3.23 ± 0.00 ^a^
***M. pulcherrima*/*S. cerevisiae***	875/898	12.70 ± 0.29 ^a^	0.01 ± 0.00 ^c^	0.02 ± 0.00 ^b^	0.17 ± 0.00 ^b^	3.45 ± 0.01 ^a^
***S. cerevisiae***	898	12.84 ± 0.26 ^a^	0.01 ± 0.00 ^c^	0.03 ± 0.01 ^b^	0.13 ± 0.01 ^b^	3.40 ± 0.02 ^a^
**Zinfandel**	Commercial strain	12.62 ± 2.25 ^a^	0.04 ± 0.00 ^a^	0.03 ± 0.00 ^b^	0.16 ± 0.02 ^b^	3.42 ± 0.01 ^a^

**Table 4:** Main volatile compounds of *S. cerevisiae* DiSVA 898 and Zinfandel pure culture and sequential fermentations of *M. pulcherrima* DiSVA 875 (Mp875/Sc 898), *H. opuntiae* DiSVA 883 (Ho883/Sc898) and *L. thermotolerans* DiSVA 890 (Lt890/Sc898). Data are means ± standard deviations. Values displaying different superscript letters (^a,b,c,d^) within each row are significantly different according to Tukey’s HSD test (*p* < 0.05).

Compound	*Sc898*	OAV	*Mp875/Sc898*	OAV	*Ho883/Sc898*	OAV	*Lt890/Sc898*	OAV	Zinfandel	OAV
**ESTERS (mg/L)**										
Ethyl butyrate	0.41 ± 0.03 ^c^	1.01	0.21 ± 0.06 ^d^	0.52	0.83 ± 0.00 ^a^	2.07	0.60 ± 0.05 ^b^	1.50	0.43 ± 0.01 ^c^	1.08
Isoamyl acetate	1.36 ± 0.01 ^cd^	8.48	1.62 ± 0.23 ^c^	10.15	2.16 ± 0.02 ^b^	13.48	3.10 ± 0.04 ^a^	19.36	1.09 ± 0.03 ^d^	6.81
Ethyl hexanoate	1.49 ± 0.03 ^a^	18.66	1.47 ± 0.10 ^a^	18.37	0.94 ± 0.01 ^b^	11.82	0.58 ± 0.04 ^c^	7.25	1.14 ± 0.06 ^b^	14.23
Hexyl acetate	0.000 ± 0.000 ^a^	0.00	0.001 ± 0.000 ^a^	0.00	0.000 ± 0.000 ^a^	0.00	0.000 ± 0.000 ^a^	0.00	0.000 ± 0.000 ^a^	0.00
Ethyl octanoate	0.01 ± 0.00 ^a^	0.02	0.01 ± 0.00 ^a^	0.02	0.01 ± 0.00 ^a^	0.02	0.01 ± 0.00 ^a^	0.01	0.01 ± 0.00 ^a^	0.02
Diethyl succinate	0.02 ± 0.00 ^a^	0.01	0.01 ± 0.00 ^b^	0.00	0.00 ± 0.00 ^b^	0.00	0.01 ± 0.00 ^b^	0.00	0.00 ± 0.00 ^b^	0.00
2-Phenyl ethyl acetate	0.07 ± 0.01 ^ab^	0.90	0.08 ± 0.01 ^a^	1.12	0.05 ± 0.00 ^ab^	0.70	0.04 ± 0.00 ^b^	0.62	0.04 ± 0.01 ^b^	0.56
Ethyl acetate	10.90 ± 4.00 ^bc^	0.91	15.29 ± 0.33 ^b^	1.27	4.86 ± 0.73 ^c^	0.41	23.69 ± 2.11 ^a^	1.97	7.75 ± 0.51 ^bc^	0.65
**CARBONYL COMPOUNDS (mg/L)**										
Acetaldehyde	46.49 ± 2.69 ^a^	92.98	33.54 ± 0.38 ^b^	67.08	16.08 ± 0.36 ^c^	32.16	43.67 ± 1.21 ^a^	87.34	29.80 ± 2.15 ^b^	59.61
**MONOTERPENS (mg/L)**										
Linalool	0.04 ± 0.01 ^ab^	2.61	0.06 ± 0.00 ^a^	3.93	0.04 ± 0.00 ^ab^	2.93	0.05 ± 0.00 ^ab^	3.58	0.04 ± 0.01 ^b^	2.55
Nerol	0.02 ± 0.01 ^a^	1.32	0.00 ± 0.00 ^a^	0.00	0.01 ± 0.00 ^a^	0.31	0.01 ± 0.00 ^a^	0.66	0.00 ± 0.00 ^a^	0.00
Geraniol	0.04 ± 0.00 ^a^	1.38	0.01 ± 0.00 ^d^	0.18	0.03 ± 0.00 ^b^	1.06	0.02 ± 0.00 ^c^	0.57	0.02 ± 0.00 ^c^	0.58
**NORISOPRENOIDS (mg/L)**										
β-damascenone	0.02 ± 0.01 ^a^	180.15	0.01 ± 0.01 ^a^	85.50	0.01 ± 0.00 ^a^	88.98	0.01 ± 0.00 ^a^	94.96	0.01 ± 0.00 ^a^	65.75
**HIGHER ALCOHOLS (mg/L)**										
Hexanol	0.25 ± 0.03 ^a^	0.03	0.19 ± 0.04 ^a^	0.02	0.32 ± 0.04 ^a^	0.04	0.22 ± 0.04 ^a^	0.03	0.24 ± 0.02 ^a^	0.03
β-Phenyl ethanol	22.08 ± 2.60 ^b^	1.58	36.92 ± 1.13 ^ab^	2.65	24.34 ± 1.07 ^ab^	1.74	39.06 ± 6.21 ^a^	2.79	29.07 ± 4.89 ^ab^	2.08
1-propanol	16.67 ± 3.21 ^ab^	0.05	12.49 ± 0.38 ^b^	0.04	14.02 ± 1.94 ^ab^	0.05	21.13 ± 0.45 ^a^	0.07	14.38 ± 2.17 ^ab^	0.05
Isobutanol	8.15 ± 4.20 ^b^	0.20	9.43 ± 0.19 ^ab^	0.24	6.89 ± 1.77 ^b^	0.17	17.32 ± 0.81 ^a^	0.43	5.29 ± 0.22 ^b^	0.13
Amyl alcohol	7.15 ± 1.76 ^a^	0.11	2.62 ± 0.50 ^a^	0.04	7.18 ± 3.45 ^a^	0.11	4.64 ± 0.12 ^a^	0.07	2.43 ± 0.14 ^a^	0.04
Isoamyl alcohol	42.93 ± 3.57 ^cd^	0.72	36.23 ± 2.12 ^d^	0.60	78.45 ± 2.55 ^a^	1.31	55.80 ± 1.58 ^b^	0.93	48.15 ± 1.49 ^bc^	0.80

OAV: odor activity value. OAV = C/OT. C (Concentration): The actual concentration of the individual compound in the sample; OT (Odor Threshold).

## Data Availability

The original contributions presented in this study are included in the article and Supplementary Materials. Further inquiries can be directed to the corresponding author.

## Supplementary Materials

The following supporting information can be downloaded at: https://www.mdpi.com/article/10.3390/foods15203706/s1, Figure S1: Interdelta PCR profiles of *Saccharomyces cerevisiae* strains obtained using δ12/δ21 primers. Marker: molecular weight marker; Blank: negative control.
